# Low serum selenium combined with SELENOP-autoantibodies are associated with persistent fatigue after SARS-CoV-2 infection

**DOI:** 10.1016/j.redox.2026.104273

**Published:** 2026-06-27

**Authors:** Börge Schmidt, Sabrina Asaad, Laven Mavarani, Andreas Stang, Thilo S. Chillon, Waldemar B. Minich, Kostja Renko, Thorsten Brenner, Lara Maria Schöler, Ulf Dittmer, Mirko Trilling, Lutz Schomburg

**Affiliations:** a Institute for Medical Informatics, Biometry and Epidemiology, University Hospital Essen, University of Duisburg-Essen, Essen, Germany; b Institute for Experimental Endocrinology, Charité - Universitätsmedizin-Berlin, Berlin, Germany; c Department of Anesthesiology and Intensive Care Medicine, University Hospital Essen, University Duisburg-Essen, Essen, Germany; d Institute for Virology, University Hospital Essen, University of Duisburg-Essen, Essen, Germany; e Institute for the Research on HIV and AIDS-associated Diseases, University Hospital Essen, University of Duisburg-Essen, Essen, Germany

## Abstract

**Background:**

Cytokines found during post-acute sequelae after COVID-19 are known to reduce selenoprotein biosynthesis, raising the question whether low selenium, impaired selenoprotein P (SELENOP) production, and autoantibodies to SELENOP (SELENOP-aAb) are associated with persistent fatigue after SARS-CoV-2 infection.

**Methods:**

Persistent symptoms and selenium determinants were assessed on average 21.9 months after a PCR-verified SARS-CoV-2 infection in a cross-sectional population-based study (n = 750 adults, 54.1% female). Study participants were categorized based on the presence of low selenium levels (<70 μg/L), low SELENOP levels (<4.1 mg/L), and SELENOP-aAb positivity (≥3.0 BI). Regression models were fitted to assess associations between selenium determinants and persistent fatigue, adjusted for potential confounders.

**Findings:**

Persistent fatigue was self-reported by 23.1% (n = 173) of participants. Low serum selenium was measured in 63.7% (n = 478), low SELENOP in 89.6% (n = 672), and elevated SELENOP-aAb in 3.9% (n = 29). A combination of low selenium and elevated SELENOP-aAb was found in 1.9% (n = 14) participants, a combination of low SELENOP and SELENOP-aAb in 3.2% (n = 24). Individually, all three selenium-related determinants showed no strong indication for an association with persistent fatigue. However, the combination of selenium deficiency and SELENOP-aAb positivity showed a prevalence ratio of 2.16 (95%-CI: 1.13-4.11), and the combination of SELENOP deficiency and SELENOP-aAb positivity a prevalence ratio of 2.15 (95% Cl: 1.01; 4.60) compared to the reference group.

**Interpretation:**

The study indicates that in a fraction of COVID-19 affected participants low selenium/SELENOP levels in combination with SELENOP-specific autoantibodies are associated with a two times higher prevalence of persistent fatigue after SARS-CoV-2 infection.

## Graphical abstract

**Figure:**
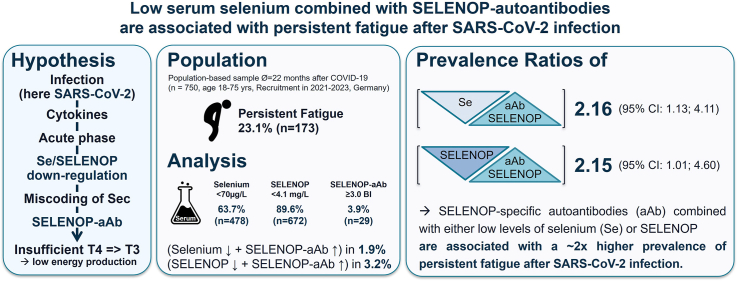

## 1 Introduction

Post-acute sequelae of COVID-19 (PASC) has been characterized by numerous symptoms that may persist after acute COVID-19 infection, commonly with conditions such as fatigue, brain fog and cognitive impairment [[1], [2], [3], [4], [5], [6], [7]]. Prevalence estimates of >10% have been described in populations after SARS-CoV-2 infection [2,8,9]. Factors promoting persistent symptoms and their severity include age, sex, preexisting health conditions and severity of the acute infection [10]. For most of the PASC-related conditions, such as persistent fatigue, current therapeutic approaches are largely symptomatic and supportive [11,12], while PASC in general has been characterized by persistent activation of chronic inflammatory pathways, suggesting new therapeutic targets [13].

The trace element selenium (Se) is essential for the biosynthesis of selenoproteins, the majority of which being redox-active enzymes containing the 21st proteinogenic amino acid selenocysteine (Sec) in their active centers [14]. Among others, the family of selenoproteins comprises five glutathione peroxidases (GPX), three thioredoxin reductases (TXNRD), and three iodothyronine deiodinases (DIO). The latter control how much active thyroid hormone (T3) is available in tissues by removing iodine atoms from thyroxine (T4) and/or T3. Another important selenoprotein is selenoprotein P (SELENOP), which contains up to ten Sec residues and mediates the Se transport to extrahepatic tissues such as endocrine glands and the brain [15,16]. Animal studies revealed that SELENOP-mediated Se transport is essentially needed under conditions of limited Se supply. In humans, low dietary Se intake impairs the capacity to synthesize selenoproteins which predisposes to increased health risks.

Based on the following chain of thoughts, we wondered if persistent fatigue after SARS-CoV-2 infection as pronounced inflammatory stimuli may in part result from an impaired Se bioavailability in tissues: (I) Both the acute SARS-CoV-2 infection and persistent fatigue are associated with pro-inflammatory cytokine signatures [[17], [18], [19], [20], [21]]. (II) Pro-inflammatory cytokines such as TNFα, IL-1β, IFNγ, and IL-6 as well as hypoxia, which is also interconnected with inflammation, reduce the biosynthesis of selenoproteins [[22], [23], [24], [25]]. This negative acute-phase response results in decreased serum Se and SELENOP levels in response to inflammation, infection, and severe disease [16]. Accordingly, a declining Se status is observed in intensive care unit patients, and mortality risk correlates to Se deficiency e.g., for COVID-19 [26]. (III) In absence of sufficient Se, Sec positions in selenoproteins can be miscoded [[27], [28], [29], [30], [31]]. (IV) Proteins harboring miscoded residues may constitute neoantigens resulting in the generation of autoantibodies. Accordingly, SELENOP-autoantibodies (SELENOP-aAb) may develop under conditions of severe inflammation, as recently shown in burn injury [32]. (V) SELENOP-aAb impair the SELENOP-mediated Se transport to extrahepatic tissues. As a consequence, the tissue Se status may decrease especially during phases of reduced Se supply, leading to reduced expression of intracellular selenoproteins. This notion was supported by data from patients with chronic fatigue, where positivity for SELENOP-aAb was associated with a specific form of hypothyroidism due to impaired activity of Se-dependent DIO [33]. Importantly, the apparent conversion defect of inactive T4 to active T3 is not reflected in aberrant thyrotropin (TSH) levels, which is the cornerstone of thyroid axis function assessments, and a more refined workup is needed to identify this type of endocrine fatigue. Based on the aforementioned rationale, the aim of the present study was to assess the potential connection of low SELENOP or impaired SELENOP activity with fatigue. To its end, we investigated the association of Se, SELENOP and SELENOP-aAb levels in a population-based sample of adults with self-reported persistent fatigue and fatigue severity >12 months after SARS-CoV-2 infection.

## 2 Methods

### 2.1 Study population

Cross-sectional data of the population-based PostCove Study were used. The PostCove Study assessed persistent symptoms, overall health status, and clinical markers for cardiovascular, metabolic, and respiratory conditions in participants >12 months after SARS-CoV-2 infection. Recruitment took place between September 2021 and May 2023. Eligible participants were residents of the City of Essen, Germany, aged 18-75 years, who had been registered by the local health authorities as having tested positive for SARS-CoV-2 infection using a polymerase chain reaction (PCR) test with the date of the first SARS-CoV-2 infection between February 2020 (the beginning of the first pandemic wave in the city of Essen) and November 2020, prior to first reports of a local circulation of the alpha variant of SARS-CoV-2. All eligible individuals were consecutively invited for participation by the local health authorities. The response proportion was 19.4%, resulting in 801 participants (54.3% female). The study was conducted in accordance with the Declaration of Helsinki and the guidelines and recommendations for ensuring Good Epidemiological Practice [34] and approved by the ethics committee of the University Duisburg-Essen (20-9628-BO). All participants gave written informed consent.

### 2.2 Data assessment

Standardized computer-assisted face-to-face interviews were conducted to assess current persistent self-perceived symptoms (including fatigue) that participants attributed to their SARS-CoV-2 infection. None of the participants reported a medical diagnosis with chronic fatigue syndrome at the time of the examination. For every persistent symptom, self-rated severity was assessed on a 1-10 numeric scale (10 = highest intensity). For some of the analyses, reporting no current persistent fatigue was coded as “0” on the severity scale or severity was further categorized as “0”, “1-5” or “6-10”. Participants were also asked to list the type and name of all COVID-19 vaccines they had received until the date of study examination. As all participants had their first SARS-CoV-2 infection at the beginning of the pandemic in 2020, the first SARS-CoV-2 infection necessarily predated the COVID-19 vaccination. Information on vaccination was coded as “any vaccine vs. no vaccination” or “any viral vector vaccine vs. no viral vector vaccine” for analysis. Education was defined by combining school and vocational training as total years of formal education according to the International Standard Classification of Education (ISCED) 2011 [35]. Blood serum samples were collected and stored at −80°C at the *Westdeutsche Biobank Essen* (WBE, University Hospital Essen, University of Duisburg-Essen, Essen, Germany).

### 2.3 Measurement of markers of selenium status

Serum samples were analysed for the levels of total Se, SELENOP, and SELENOP-aAb, essentially as has been described previously [33,36]. Briefly, aliquots were diluted with a gallium-standard (1.0 mg/L, Alfa Aesar GmbH, Karlsruhe, Germany) and applied to polished glass carriers. After drying, samples were subjected to total reflection X-ray fluorescence (TXRF) by a TXRF analyzer (S4 T-STAR, Bruker Nano GmbH, Berlin, Germany). A serum standard (Seronorm, Sero AS, Billingstad, Norway) served control purposes. The measured levels were within the specified range of the standard, and the inter-assay coefficient of variation (CV) was <5% during the analyses. SELENOP was determined by a chemiluminescent immunoassay using an automated system (iSYS automat, Immunodiagnostic Systems Holdings Ltd, Frankfurt, Germany), with monoclonal antibodies against SELENOP as described [37]. Three samples spanning the working range of the assay were used for control purposes (selenOmed GmbH, Berlin, Germany). The inter- and intraassay CV were determined to be below 10% during the analyses. Levels of SELENOP-aAb were determined by a precipitation assay using protein A (ASKA Biotech GmbH, Berlin, Germany) and commercial standards (selenOmed GmbH). Serum samples were incubated with a fusion protein of recombinant SELENOP coupled to secreted alkaline phosphatase (SEAP). Following addition of protein A, immune complexes were precipitated by centrifugation, washed, and the bound antibody-antigen-reporter complexes were quantified by assessing SEAP activity using a luminometer (Berthold Technologies GmbH, Bad Wildbad, Germany). A binding index (BI) of each sample was calculated as factor of the relative light units (RLU) obtained divided by the average RLU of negative control samples (BI = 1.0), as described earlier [33]. A sensitive cut-off for positive autoimmunity to SELENOP of BI ≥ 3.0 was chosen as described [32], Se deficiency was classified when serum Se levels were <70 μg/L, in agreement with other large European studies [38], and SELENOP deficiency was classified at serum levels <4.1 mg/L, as determined in the largest study on its association with overall mortality [39].

### 2.4 Statistical analysis

Of the 801 participants in the PostCove Study, 750 had non-missing information on persistent fatigue and Se status (Fig. S1). Histograms and box plots were generated to visualize the distribution of Se, SELENOP and log_e_(SELENOP-aAb) levels, stratified by the current presence of fatigue and by fatigue severity category. Log-binomial regression models were fitted to assess the association of Se, SELENOP, and SELENOP-aAb with the presence of current persistent fatigue, adjusted for the potential confounders age, sex, months between first infection and study examination, education, and any viral vector vaccine, to estimate prevalence ratios and corresponding 95% confidence intervals (95%-CIs). The confounders were selected by using a directed acyclic graph (DAG) to derive a minimal adjustment set for estimating the total effect. Linear regression models were fitted to assess the association of Se, SELENOP, and SELENOP-aAb with severity of current persistent fatigue, adjusted for the same potential confounders, to estimate beta estimates (β) and corresponding 95% CIs. Effect measures were presented per interquartile range (IQR) of Se, SELENOP and SELENOP-aAb, respectively.

To assess whether a combination of low Se or low SELENOP with high SELENOP-aAb levels was associated with presence or severity of current persistent fatigue, participants were stratified into four categories each according to Se, SELENOP and SELENOP-aAb cut-offs. These categorical variables were included separately using dummies with the categories of low Se / SELENOP and high SELENOP-aAb as reference. All statistical analyses were performed using SAS software, version 9.4 (SAS Institute Inc., Cary, NC, USA). Results from the log-binomial regressions were visualized using RStudio (version 2025.05.1 Build 513) with R (version 4.4.1; Posit Software, PBC).

## 3 Results

The mean age of the analysis population was 48.3 ± 14.1 years, and 406 (54.1%) participants were female (Table 1). The mean time between SARS-CoV-2 infection and the examination date was 21.9 ± 4.0 month. Overall, 701 (93.5%) participants were vaccinated after SARS-CoV-2 infection. Of these, 134 (17.9%) had received a viral vector vaccine. Median selenium was 66.6 (IQR: 59.7; 73.8) μg/L, and median SELENOP was 3.1 (IQR: 2.6; 3.7) mg/L. SELENOP-aAb levels had a median of 0.7 (IQR: 0.5; 0.9) BI. Fatigue was reported by 173 (23.1%) participants. There were 14 (1.9%) participants with both low selenium and high SELENOP-aAb levels, while 24 (3.2%) participants had both low SELENOP and high SELENOP-aAb levels.

**Table 1:** Characteristics of the study population (n = 750).

Covariates of interest	n = 750
Sex (female)	406 (54.1%)
Age (years)	48.3 ± 14.1
Selenium (μg/L)	66.6 (59.7; 73.8)
SELENOP (mg/L)	3.1 (2.6; 3.7)
SELENOP-aAb (BI)	0.7 (0.5; 0.9)
Log_e_(SELENOP-aAb)	0.5 (0.4; 0.6)
Selenium ≥70 μg/L and SELENOP-aAb <3.0 BI	257 (34.3%)
Selenium <70 μg/L and SELENOP-aAb <3.0 BI	464 (61.9%)
Selenium ≥70 μg/L and SELENOP-aAb ≥3.0 BI	15 (2.0%)
Selenium <70 μg/L and SELENOP-aAb ≥3.0 BI	14 (1.9%)
SELENOP ≥4.1 mg/L and SELENOP-aAb <3.0 BI	73 (9.7%)
SELENOP <4.1 mg/L and SELENOP-aAb <3.0 BI	648 (86.4%)
SELENOP ≥4.1 mg/L and SELENOP-aAb ≥3.0 BI	5 (0.7%)
SELENOP <4.1 mg/L and SELENOP-aAb ≥3.0 BI	24 (3.2%)
Time since SARS-CoV-2 infection (months) missings = 3	21.9 ± 4.0
Any COVID-19 vaccine	701 (96.4%)
Viral vector vaccine	134 (17.9%)
Education (years)	16 (13; 17)
Persistent fatigue (yes)	173 (23.1%)
Fatigue severity (in participants reporting fatigue; n = 172) missings = 1	5.5 ± 2.3
Fatigue severity 0 (no fatigue)	577 (77.0%)
Fatigue severity 1 to 5	89 (11.9%)
Fatigue severity 6 to 10	83 (11.1%)

Continuous variables as mean ± SD or median (Q1; Q3); categoric variables as frequency (%).

SELENOP; selenoprotein P, Se; selenium, BI; binding index.

**Fig. 1:**
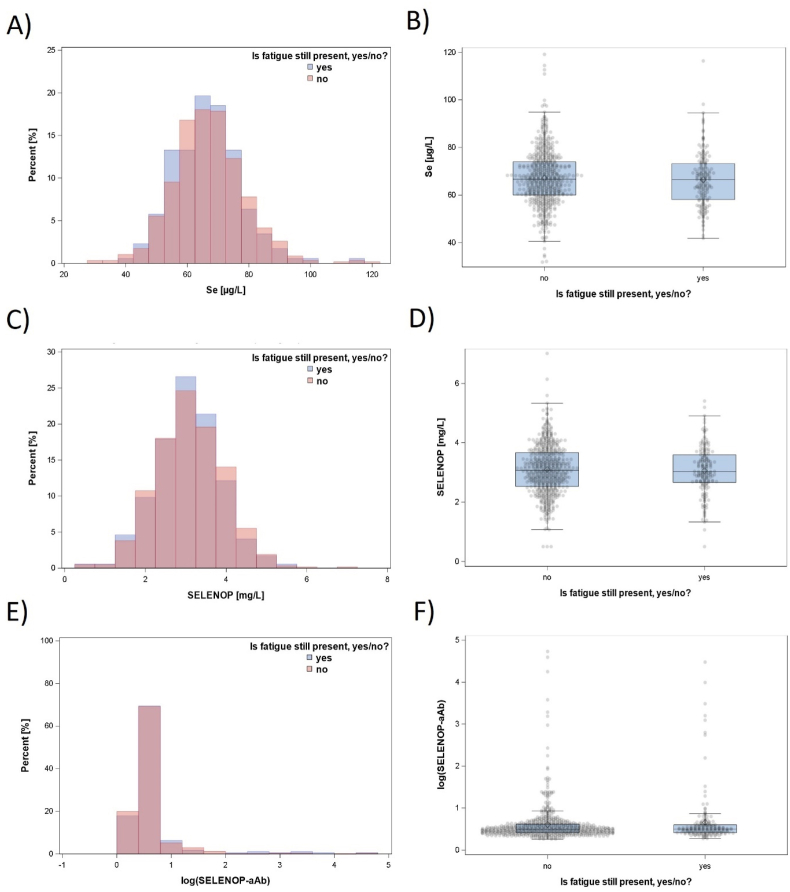
Histograms and boxplots for the distribution of selenium (Se) (A, B), SELENOP (C, D) and log_e_(SELENOP-aAb) (E, F) stratified by the current presence of fatigue (n = 750).

Histograms for Se and SELENOP stratified by fatigue status indicated a near-symmetric distribution for both groups with a slight shift towards higher levels for the group without current persistent fatigue (Fig. 1A and C). Corresponding box plots (Fig. 1B and D) showed comparable median levels for Se (66.5 μg/L, IQR: 58.1; 73.2 and 66.6 μg/L, IQR: 60.0; 74.0, respectively), but a small difference for SELENOP with slightly lower levels in participants with fatigue (3.03 mg/L, IQR: 2.66; 3.59 vs. 3.07 mg/L, IQR: 2.53; 3.67). Histograms for log_e_(SELENOP-aAb) stratified by fatigue status showed no strong differences in the distribution between groups (Fig. 1E). The corresponding box plots (Fig. 1F) also indicated no difference between median levels of SELENOP-aAb (0.50, IQR: 0.41; 0.60 and 0.50, IQR: 0.41; 0.62, respectively). Boxplots for Se and SELENOP stratified by fatigue severity (Fig. S2a and S2b), grouped as 0 (no fatigue), severity categorized as 1-5, and severity categorized as 6-10, showed lower median levels of Se and SELENOP in the group reporting high severity (Se: 63.4 μg/L, IQR: 55.2; 71.0; SELENOP: 2.93 mg/L, IQR: 2.44; 3.45) compared to the lower severity group (Se: 67.8 μg/L, IQR: 62.0; 73.7; SELENOP: 3.22 mg/L, IQR: 2.72; 3.70). Boxplots for log_e_(SELENOP-aAb) stratified by fatigue severity showed comparable median levels between the groups of different fatigue severity (Fig. S2c). The frequency of persistent fatigue stratified by Se / SELENOP-aAb and SELENOP / SELENOP-aAb categories showed the highest prevalence in the group with low Se and high SELENOP-aAb (Table S1).

There was only a weak indication for an association of lower Se and SELENOP levels alone with a higher prevalence of persistent fatigue (Fig. S3). In the log-binomial regression model adjusted for the potential confounders age, sex, months after infection, education and viral vector vaccination, the presence of fatigue was 0.92-fold lower (95% CI: 0.79 – 1.08) per IQR of Se and 0.90-fold lower (95% CI: 0.76 – 1.07) per IQR of SELENOP. Using fatigue severity in participants reporting persistent fatigue as outcome in linear regression models, an association with Se was observed indicating a 0.56 point (95% CI: −0.98; −0.14) lower severity per Se IQR (Table S2). For SELENOP-aAb alone, no strong indication for an association with persistent fatigue was observed (Fig. S3). The prevalence ratio showed only a slightly higher prevalence of fatigue per IQR of SELENOP-aAb. No association was observed for SELENOP-aAb when using fatigue severity as outcome (Table S2).

**Fig. 2:**
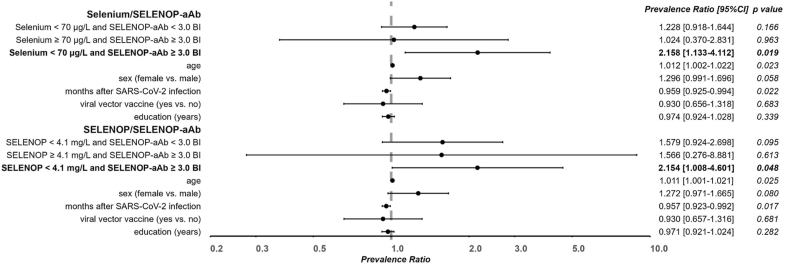
Prevalence ratios (PR) and 95% confidence intervals (Cl) from log-binominal regression models for the association of selenium/SELENOP-aAb categories (category Se ≥ 70 μg/L and SELENOP-aAb <3.0 BI as reference) and SELENOP/SELENOP-aAb categories (category SELENOP ≥4.1 mg/L and SELENOP-aAb <3.0 BI as reference) with persistent fatigue adjusted for age, sex, education, months after SARS-CoV-2 infection, and viral vector vaccine (n = 747).

Next, in a confounder adjusted log-binomial regression model, the association of Se / SELENOP-aAb categories with persistent fatigue was analysed using the group with Se ≥ 70 μg/L and SELENOP-aAb <3.0 BI as reference. There was a 2.16-fold (95% Cl: 1.13; 4.11) higher prevalence of fatigue in the group with low Se and high SELENOP-aAb levels, compared to the reference group (Fig. 2). The prevalence ratio for the group with low Se but negative SELENOP-aAb assessment gave only weak indication for a moderately higher fatigue prevalence compared to the reference group. No association for the group with normal Se and positivity of SELENOP-aAb levels was observed. In linear regression models using fatigue severity as outcome, there was no indication for an association with Se / SELENOP-aAb (Table S3).

Results for the association of SELENOP / SELENOP-aAb categories with persistent fatigue were consistent to the findings using Se / SELENOP-aAb categories (Fig. 2): there was strong indication for a higher prevalence of fatigue in the group with low SELENOP and high SELENOP-aAb levels with an adjusted prevalence ratio of 2.15 (95% Cl: 1.01; 4.60) compared to the reference group. In linear regression models using fatigue severity as outcome again no indication for an association was observed (Table S4).

In all regression models (i.e., Fig. 2 and S3), higher age and female sex showed associations with higher prevalence of current persistent fatigue, while with increasing time between SARS-CoV-2 infection and examination date fatigue prevalence was lower. Higher years of education gave weak indication for a lower fatigue prevalence. Having received viral vector vaccination was not associated with fatigue prevalence.

## 4 Discussion

The present study investigated the association between Se / SELENOP deficiency in combination with the presence of SELENOP-aAb and persistent fatigue after SARS-CoV-2 infection. The results indicated that a certain fraction of patients with persistent fatigue following a preceding SARS-CoV-2 infection were affected by low Se, low SELENOP, and SELENOP-aAb in a population-based study sample. While serum levels of Se, SELENOP, or SELENOP-aAb alone showed no strong associations in confounder-adjusted regression analyses, participants with Se deficiency (<70 μg/L) or low SELENOP (<4.1 mg/L) in combination with positivity for SELENOP-aAb (BI ≥ 3.0) had a more than 2-fold higher prevalence of persistent fatigue. Potential confounders such as higher age, female sex and lower education, showed associations with persistent fatigue in the direction expected. The overall findings support the hypothesis of a dysregulated Se metabolism as a relevant aspect of chronic fatigue in a subset of patients.

Persistent fatigue is a hard-to-quantify diagnosis with a multitude of potential underlying reasons and a wide spectrum of associated symptoms. The incidence increased dramatically after the COVID-19 pandemic, and different mechanisms are discussed as potential underlying cause for the severity of this kind of post-infection syndrome, including new-onset autoimmune disease, re-activation of virus, persistence of viral particles or long-term inflammation [12]. Fatigue is diagnosed more frequently in subjects with pre-existing autoimmune disease, supporting the notion that autoimmunity might be involved [40]. The rationale for assessing the Se status in relation to persistent fatigue was based on the clinical picture described for patients with chronic fatigue syndrome, low T3-/non-thyroidal illness syndrome and Hashimoto's thyroiditis, assuming an endocrine disturbance of thyroid hormone activation as a central aspect of the over-arching symptoms [41]. Thyroid hormone is essentially needed for energy homeostasis in all cells, supporting a sufficiently high basal metabolic rate, ATP synthesis and physical and mental strength [42]. Hypothyroidism causes symptoms of low energy, reduced metabolism, weakness, obstipation, mental disturbance, cold intolerance and suppressed quality of life [43]. Clinically, hypothyroid patients display increased TSH levels, enabling a straightforward diagnosis and therapeutic correction by thyroid hormone substitution to achieve TSH reference levels. When a patient is analysed and TSH is found within the reference range, no further diagnosis for hypothyroidism or therapeutic adaptation is routinely made. Yet, certain patients may suffer from a conversion defect where activation of the prohormone T4 to T3 is impaired in tissues without a respective increase in serum TSH, due to inherited polymorphisms in relevant genes [44], high inflammation in severe disease (low-T3-/non-thyroidal illness syndrome) [45], or mutations in central components of the selenoprotein biosynthesis machinery (e.g., in SECIS-binding protein 2, SECISBP2) [46]. Hence, tissue hypothyroidism with low T3 levels may develop despite normal serum TSH levels under certain conditions, causing fatigue symptoms. Accordingly, the potential dysregulation of the thyroid axis was studied in CFS patients in detail, and a resemblance to low-T3-/non-thyroidal illness was reported with a characteristic reduction in T3 and a particular increase in rT3, an inactive metabolite of T4 ^41^. This pattern accorded to patients with inherited defects in selenoprotein biosynthesis, where DIO expression was impaired, and low T3, elevated rT3 and normal TSH levels were determined [46]. But besides this genetic impairment, also Se transport defects due to SELENOP-aAb have been described with a similar thyroid hormone pattern [36]. An acquired autoimmune issue due to newly developed autoantibodies is also a consistent observation after COVID-19 [47].

While the impact of low Se status and SELENOP-aAb positivity due to SARS-CoV-2 infection on developing persistent fatigue appears plausible, the cross-sectional design of the present study does not allow distinguishing causal effect from reverse causation. Low Se status is a general risk factor for autoimmunity, as documented in large studies in central China, where both prevalence and incidence of Hashimoto's thyroiditis was higher in areas with habitual low Se intake as compared to neighbouring areas with a better Se supply [48,49]. However, low Se may also develop in response to disease, as typically observed on the ICU under severe inflammation [50]. The same applies to SELENOP-aAb, as they may develop in response to an inflammatory disease and predispose to a severe disease course [32]. This has been shown after burn injury, where SELENOP-aAb newly appeared around one months after the accident, and in breast cancer, where the mortality rate of SELENOP-aAb positive cases was particularly increased under conditions of Se deficiency [51]. The finding that Se and SELENOP levels in the present study were on average lower compared to previous reports of pre-pandemic population-based study samples [52,53] might be a direct result of the SARS-CoV-2 infection.

Among the particular strengths of this analysis was the geographical separation of the study site and the analytical labs, and that all the involved personnel remained blinded during the laboratory analyses until data collection was completed. Another strength was that important confounders were available for analysis. Education was included in the adjustment set as indicator for health-related risks associated with socioeconomic position that may have simultaneously affected Se status and presence of persistent fatigue such as comorbidities, dietary and other lifestyle factors associated with inflammation. However, even after adjustment for important confounders there may still be residual confounding not accounted for, which is a limitation of this study. An additional strength was that the study enrolment used a population-based recruitment strategy during early stages of the COVID-19 pandemic from a well-defined study region. While this study design led to a sample reflecting the Se status and outcome distribution after infection with early SARS-CoV-2 variants in the source population (i.e., general population), the number of participants with impaired Se status and severe fatigue was expected to be rather small. However, statistical power was sufficient for detecting moderate to strong associations between Se status and persistent fatigue with the given sample size, exposure and outcome prevalence.

In conclusion, an impaired systemic Se supply characterized by low circulating concentrations of Se and/or SELENOP in combination with autoimmunity to Se transport, i.e., SELENOP-aAb, associates with persistent fatigue after SARS-CoV-2 infection. This specific trace element deficiency likely results from a combination of poor nutritional intake, inflammation and autoimmunity, and may constitute a preventable and addressable issue, as intervention trials with supplemental Se have proven effective in the areas of consistent endemic Se deficiency [54]. Without actively counteracting the impaired Se status, it appears unlikely that it resolves by itself as Europe constitutes an area of limited Se availability with low nutritional uptake, and post-infection inflammation impairs regular selenoprotein biosynthesis, closing a vicious cycle of deficiency. While it thus appears as an addressable condition, it only applies to a small fraction of affected individuals, and does not constitute the only and most prevalent metabolic, immune or endocrine derangement. However, even if we assume a conservative incidence of chronic fatigue after SARS-CoV-2 infection in the general population [[55], [56], [57]], the number of individuals affected by Se / SELENOP deficiency in combination with the presence of SELENOP-aAb would still be in the millions given the high number of confirmed SARS-CoV-2 infections worldwide. Yet, it merits consideration, diagnostic workup and eventually nutritional or supplemental correction, to enable normalization and improve recovery odds of affected patients.

## Funding

The study was funded by the German Research Council.

## CRediT authorship contribution statement

**Börge Schmidt:** Conceptualization, Data curation, Formal analysis, Funding acquisition, Investigation, Project administration, Supervision, Visualization, Writing – original draft, Writing – review & editing. **Sabrina Asaad:** Data curation, Methodology, Validation, Writing – original draft, Writing – review & editing. **Laven Mavarani:** Formal analysis, Visualization, Writing – original draft, Writing – review & editing. **Andreas Stang:** Data curation, Funding acquisition, Writing – review & editing. **Thilo S. Chillon:** Writing – review & editing. **Waldemar B. Minich:** Writing – review & editing. **Kostja Renko:** Writing – review & editing. **Thorsten Brenner:** Writing – review & editing. **Lara Maria Schöler:** Data curation, Writing – review & editing. **Ulf Dittmer:** Writing – review & editing. **Mirko Trilling:** Conceptualization, Data curation, Investigation, Supervision, Visualization, Writing – original draft, Writing – review & editing. **Lutz Schomburg:** Conceptualization, Data curation, Investigation, Methodology, Supervision, Validation, Writing – original draft, Writing – review & editing.

## Declaration of competing interest

The authors declare the following financial interests/personal relationships which may be considered as potential competing interests: Lutz Schomburg reports a relationship with selenOmed GmbH that includes: equity or stocks. If there are other authors, they declare that they have no known competing financial interests or personal relationships that could have appeared to influence the work reported in this paper.

## Data Availability

Due to data security reasons (i.e., data contain potentially participant identifying information), the PostCove Study does not allow sharing data as a public use file. However, others can access the data used upon request, which is the same way authors of the present paper obtained the data. Data requests can be addressed to: recall@uk-essen.de.
