# Supplementary material for: Low serum selenium combined with SELENOP-autoantibodies are associated with persistent fatigue after SARS-CoV-2 infection
Source: Redox Biol. 2026 Jun 27;95:104273. doi: 10.1016/j.redox.2026.104273 (PMC13334397; doi:10.1016/j.redox.2026.104273)
Supplement: Multimedia component 1 [file mmc1.docx]

**Supplement**


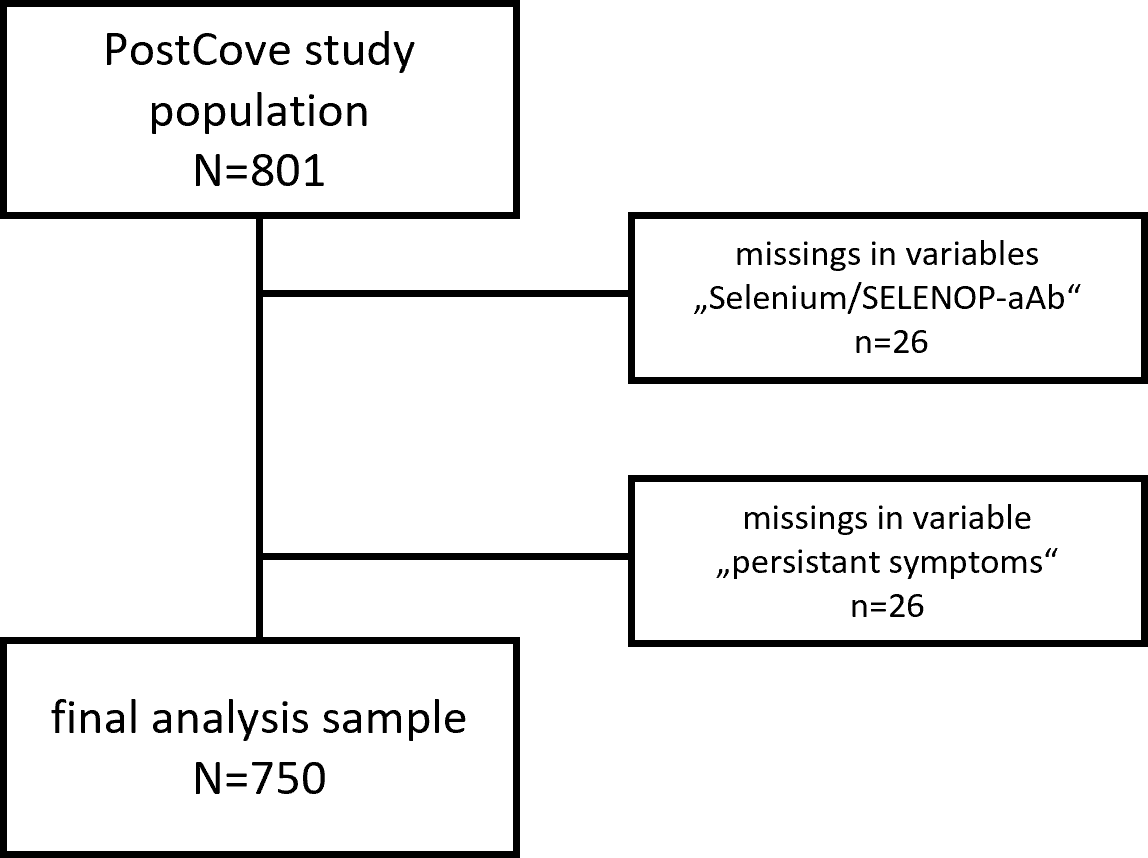


**Figure S1:** Flow chart of participants included in the analysis.


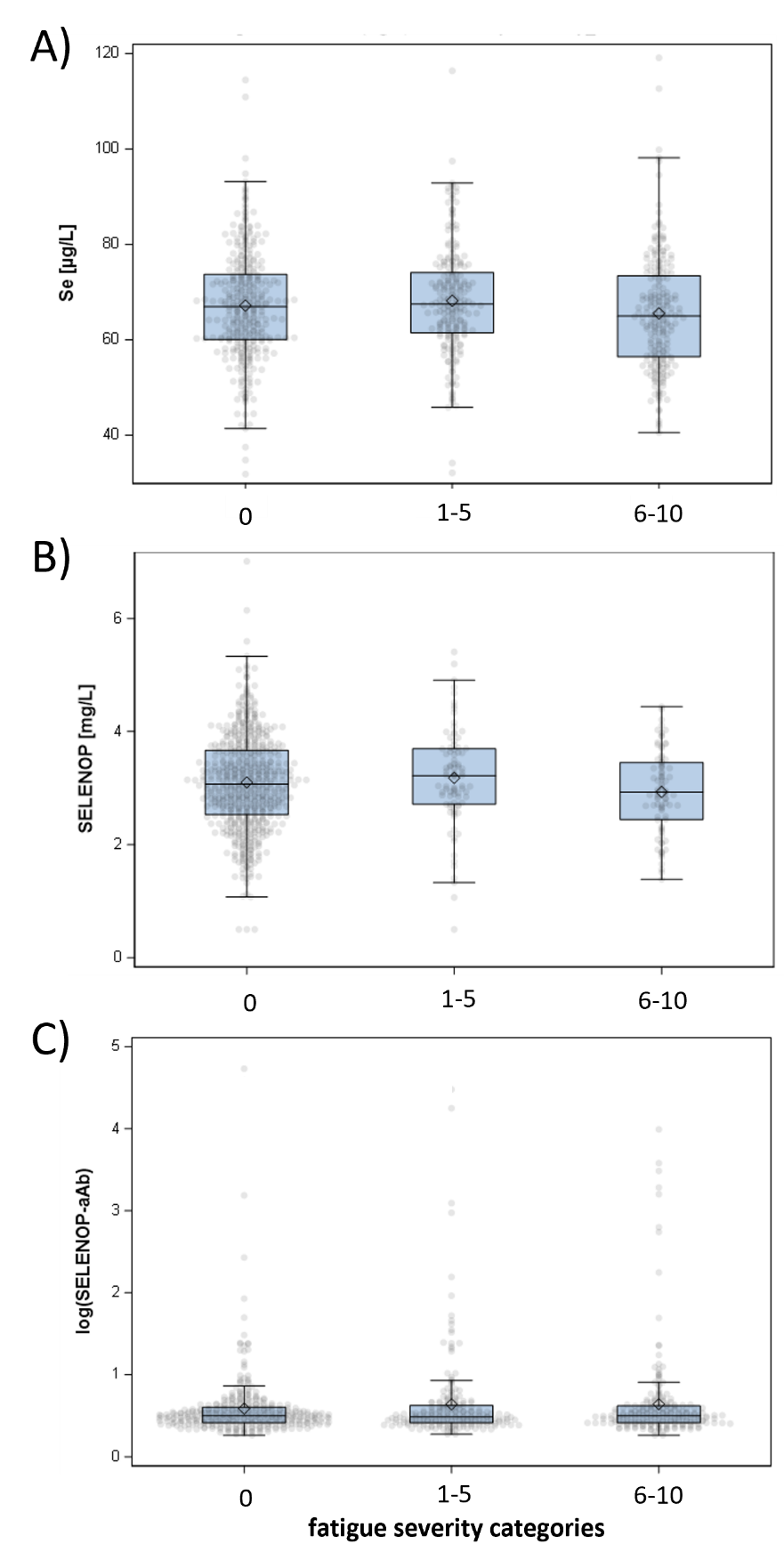


**Figure S2:** Boxplots for Selenium (Se) (A), SELENOP (B), and SELENOP-aAb (C) distribution stratified by self-rated fatigue severity (0= no fatigue, 1-5= moderate severity, 6-10= high severity; n=749).

**Table S1** Prevalence of persistent fatigue stratified by Se/SELENOP-aAb and SELENOP/SELENOP-aAb category (n=750).

| **Selenium/SELENOP-aAb categories** | **N** | **n fatigue (%)** |
| --- | --- | --- |
| Selenium ≥ 70 µg/L and SELENOP-aAb BI < 3.0 | 257 | 52 (20.2%) |
| Selenium < 70 µg/L and SELENOP-aAb BI < 3.0 | 464 | 111 (23.9%) |
| Selenium ≥ 70 µg/L and SELENOP-aAb BI ≥ 3.0 | 15 | 4 (26.7%) |
| Selenium < 70 µg/L and SELENOP-aAb BI ≥ 3.0 | 14 | 6 (42.9%) |
| **SELENOP/SELENOP-aAb categories** | **N** | **n fatigue (%)** |
| SELENOP ≥ 4.1 mg/L and SELENOP-aAb BI < 3.0 | 73 | 12 (16.4%) |
| SELENOP < 4.1 mg/L and SELENOP-aAb BI < 3.0 | 648 | 151 (23.3%) |
| SELENOP ≥ 4.1 mg/L and SELENOP-aAb BI ≥ 3.0 | 5 | 2 (40.0%) |
| SELENOP < 4.1 mg/L and SELENOP-aAb BI ≥ 3.0 | 24 | 8 (33.3%) |


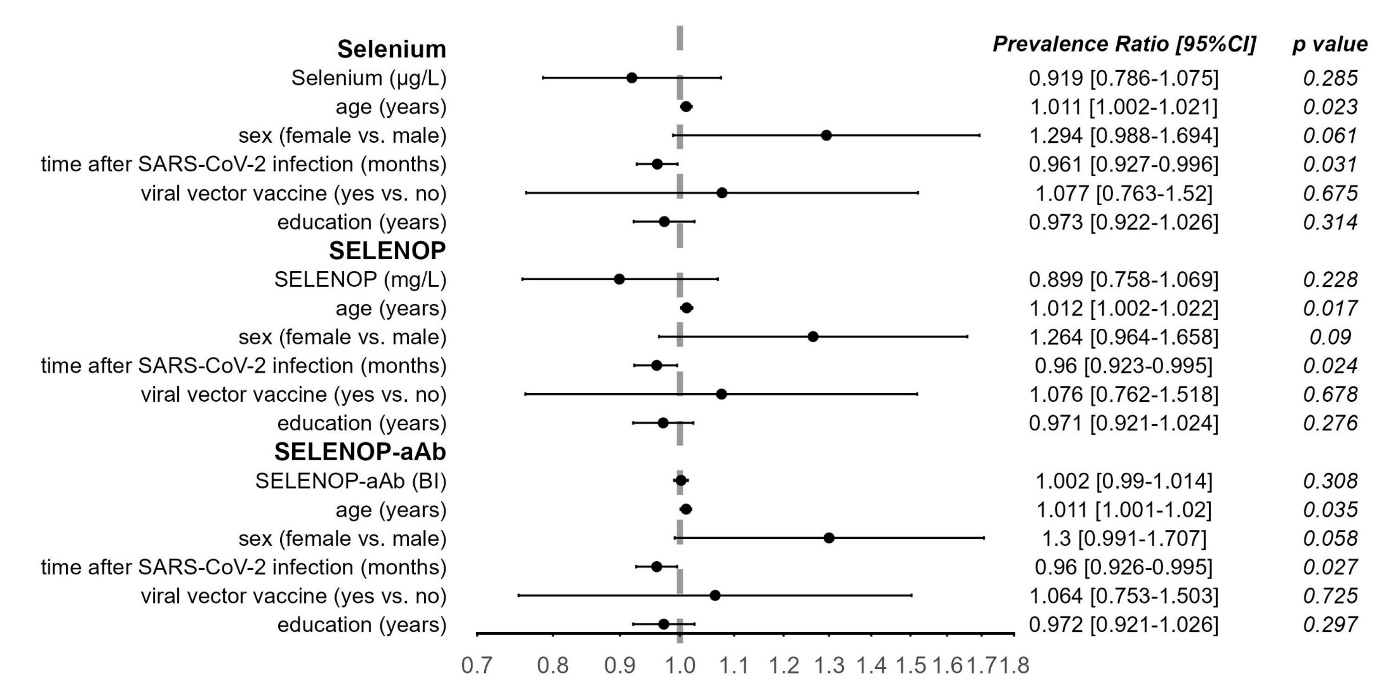
**Figure S3:** Prevalence ratios (PR) and 95% confidence intervals (Cl) per interquartile range (IQR) from log-binominal regression models for the association of Se, SELENOP and SELENOP-aAb with persistent fatigue, adjusted for age, sex, months after SARS-CoV-2 infection, years of education and viral vector vaccine (n=747).

**Table S2:** Beta estimates and 95% confidence intervals (Cl) per interquartile range (IQR) from linear regression models for the association of Selenium, SELENOP, and SELENOP-aAb with self-rated fatigue severity, adjusted for age, sex, months after SARS-CoV-2 infection, years of education and viral vector vaccine not including participants without fatigue (n=171).

| **Outcome: fatigue severity** | **Beta (95% Cl)** | **p value** |
| --- | --- | --- |
| Selenium (µg/L) | -0.561 (-0.981;-0.141) | 0.010 |
| Age (years) | -0.014 (-0.041;0.013) | 0.315 |
| Sex (female vs. male) | 0.959 (0.263;1.655) | 0.007 |
| Time since SARS-CoV-2 infection (months) | 0.043 (-0.037;0.123) | 0.289 |
| Viral vector vaccine (yes vs. no) | -0.460 (-1.327;0.407) | 0.296 |
| Education (years) | -0.085 (-0.234;0.064) | 0.259 |
| SELENOP (mg/L) | -0.316 (-0.783;0.152) | 0.188 |
| Age (years) | -0.013 (-0.042;0.015) | 0.361 |
| Sex (female vs. male) | 0.839 (0.129;1.549) | 0.021 |
| Time since SARS-CoV-2 infection (months) | 0.034 (-0.047;0.115) | 0.406 |
| Viral vector vaccine (yes vs. no) | -0.379 (-1.257;0.498) | 0.395 |
| Education (years) | -0.101 (-0.252:0.050) | 0.187 |
| SELENOP-aAb (BI) | -0.002 (-0.016;0.012) | 0.772 |
| Age (years) | -0.017 (-0.045;0.035) | 0.236 |
| Sex (female vs. male) | 0.898 (0.190;1.607) | 0.013 |
| Time since SARS-CoV-2 infection (months) | 0.040 (-0.042;0.121) | 0.337 |
| Viral vector vaccine (yes vs. no) | -0.365 (-1.250;0.519) | 0.416 |
| Education (years) | -0.114 (-0.265;0.036) | 0.136 |

**Table S3:** Beta estimates and 95% confidence intervals (Cl) from linear regression models for the association of Selenium/SELENOP-aAb categories (category Selenium ≥ 70 µg/L and SELENOP-aAb BI < 3.0 as reference) with self-rated fatigue severity, adjusted for age, sex, months after SARS-CoV-2 infection, years of education and viral vector vaccine not including participants without fatigue (n=171).

| **Outcome: fatigue severity** | **Beta (95% Cl)** | **p value** |
| --- | --- | --- |
| Selenium < 70 µg/L and SELENOP-aAb < 3.0 BI | 0.317 (-0.438;1.072) | 0.409 |
| Selenium ≥ 70 µg/L and SELENOP-aAb ≥ 3.0 BI | -1.103 (-3.755;1.549) | 0.413 |
| Selenium < 70 µg/L and SELENOP-aAb ≥ 3.0 BI | -0.141 (-2.043;1.762) | 0.884 |
| Age (years) | -0.015 (-0.043;0.013) | 0.289 |
| Sex (female vs. male) | 0.961 (0.244;1.678) | 0.009 |
| Time since SARS-CoV-2 infection (months) | 0.047 (-0.035;0.129) | 0.264 |
| Viral vector vaccine (yes vs. no) | 0.405 (-0.483;1.292) | 0.369 |
| Education (years) | -0.111 (-0.264; 0.041) | 0.152 |

**Table S4:** Beta estimates and 95% confidence intervals (Cl) from linear regression models for the association of SELENOP/SELENOP-aAb categories (category SELENOP ≥ 4.1 mg/L and SELENOP-aAb < 3 BI as reference) with self-rated fatigue severity, adjusted for age, sex, months after SARS-CoV-2 infection, years of education and viral vector vaccine not including participants without fatigue (n=171).

| **Outcome: fatigue severity** | **Beta (95% Cl)** | **p value** |
| --- | --- | --- |
| SELENOP < 4.1 mg/L and SELENOP-aAb < 3.0 BI | 0.574 (-0.788;1.936) | 0.406 |
| SELENOP ≥ 4.1 mg/L and SELENOP-aAb ≥ 3.0 BI | -3.130 (-7.727;1.467) | 0.181 |
| SELENOP < 4.1 mg/L and SELENOP-aAb ≥ 3.0 BI | 0.270 (-1.793;2.332) | 0.797 |
| Age (years) | -0.016 (-0.044;0.012) | 0.258 |
| Sex (female vs. male) | 0.865 (0.136;1.593) | 0.020 |
| Time since SARS-CoV-2 infection (months) | 0.033 (-0.048;0.115) | 0.421 |
| Viral vector vaccine (yes vs. no) | 0.393 (-0.490;1.276) | 0.381 |
| Education (years) | -0.102 (-0.252; 0.049) | 0.186 |
